# Polyphenolic Compound Variation in Globe Artichoke Cultivars as Affected by Fertilization and Biostimulants Application

**DOI:** 10.3390/plants11152067

**Published:** 2022-08-08

**Authors:** Vincenzo Montesano, Donatella Negro, Gabriella Sonnante, Gaetano Laghetti, Marcella Urbano

**Affiliations:** 1 URT-ALSIA, Institute for Sustainable Plant Protection (IPSP), National Research Council (CNR), S.S. 106 Km 448,000, 75012 Bernalda, Italy; 2 Institute of Biosciences and BioResources (IBBR), National Research Council (CNR), Via Amendola, 165/A, 70126 Bari, Italy

**Keywords:** globe artichoke, nitrogen fertilization, biostimulants, phenolic compound, HPLC-DAD-MS/MS

## Abstract

Globe artichoke is an ancient herbaceous plant native to the Mediterranean Basin. The edible part of the plant is limited to the fleshy leaves (bracts) and receptacle of a large immature inflorescence (head) that has been shown to be a rich source of bioactive compounds. Nutritional and pharmacological properties of artichoke heads and leaves are attributed mainly to polyphenolic compounds and inulin present at high concentration. In this study, polyphenols were investigated in two artichoke cultivars (Opal and Madrigal) in response to four nitrogen rates and foliar applications of biostimulating products under drip irrigation. Field experiments were carried out over two growing seasons (2015–2016, 2016–2017) in Policoro (MT), Southern Italy, on a deep clay soil in sub-humid climate conditions. Phenolic compounds were isolated and characterized by means of high-performance liquid chromatography with photodiode array detection and electrospray ionization/mass spectrometry (HPLC-DAD-MS/MS) analysis. In both cultivars, caffeoylquinic acids were more abundant when a dose of 100 kg ha^−1^ of ammonium nitrate was provided, whereas apigenins were not affected by nitrogen fertilization. Luteolins increased in cv Opal and decreased in cv Madrigal following N fertilization. The application of biostimulants (3 L ha^−1^) favored the accumulation of polyphenols, in particular of caffeoylquinic acids and apigenin, in artichoke heads in both cultivars. The results obtained highlight some positive aspects related to the synergistic effect of nitrogen fertilization and biostimulant foliar application.

## 1. Introduction

*Cynara cardunculus* var. *scolymus* (L.) Fiori, commonly known as the globe artichoke, is a vegetable characteristic of the Mediterranean area belonging to the Asteraceae family [1]. Since its ancient origins, the globe artichoke has contributed significantly to the Mediterranean agricultural economy, and Italy, in time, has become the main world producer [2] with an annual production of 378,820 tons [3].

The artichoke immature flower heads are appreciated for their organoleptic characteristics and in recent years, the interest has increased also for their richness in nutraceutical [1,4,5,6,7] and mineral compounds [8,9,10]. Since ancient times, and especially nowadays that the Mediterranean diet has been recognized as intangible Cultural Heritage of Humanity [11], the globe artichoke has also been used as herbal medicine thanks to its beneficial and therapeutic effects [12].

Polyphenols are widespread constituents of plants that have been used in the treatment of diseases for centuries. Consumption of the phytochemicals present in fruits and vegetables, particularly phenolic compounds, has been linked to reduced risk of coronary heart diseases, neurodegenerative diseases, and certain forms of cancers [13,14,15]. Artichoke extracts have surprising pharmacological and biochemical effects, such as a marked antioxidative potential and cancer chemo-preventive properties [16,17,18].

Many studies have focused on artichoke antioxidant properties but few of these are related to the abundance of the polyphenolic fractions in head parts or in various developmental stages of the plant [7,8,19,20], particularly if these compounds are influenced by agricultural practices. Regarding polyphenols, their concentrations in artichoke can be influenced by several factors, such as genotypes, physiological stages, agro-technical processes, and the different parts considered (receptacle, inner and outer bracts, and leaves) [21].

Globe artichoke leaf extracts have been widely used in herbal medicine as hepatoprotectors and choleretics [1,7], and also possess anticarcinogenic, anti-HIV, antioxidative, diuretic, as well as antifungal and antibacterial properties [7,17]. The beneficial effects of artichoke plant on human health are mainly due to flavonoids and phenolic acids, particularly caffeic acid and its derivatives mono and dicaffeoylquinic acids.

Italy encompasses an extraordinary richness in artichoke variable germplasm [22,23], and some studies have been carried out for understanding genotype contribution to polyphenol content [23].

The present work reports on the evaluation of polyphenolic compounds in artichoke heads of two artichoke varieties, in response to different nitrogen rates and foliar applications of biostimulating products under drip irrigation. The aim of this research is to determine the influence of increasing doses of nitrogen (0, 50, 100 and 150 kg ha^−1^) applied on the ground as ammonium nitrate (NH_4_NO_3_) and biostimulants (0 and 3 L ha^−1^) applied by foliar spraying as seaweed cream, on the concentration of polyphenolic compounds from the extracts of cv. Opal and Madrigal heads, subdivided in external, intermediate, internal bracts, and receptacle.

## 2. Results and Discussion

This study allowed us to carry out a quantitative evaluation of the polyphenolic profile of the heads of two artichoke cultivar in response to different nitrogen and biostimulant rates. Polyphenols were measured for three bract orders (external, intermediate, internal) and for the receptacle.

**Figure 1:**
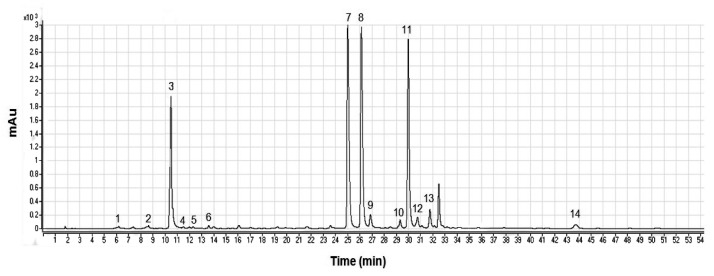
Separation of caffeoylquinic acids and flavonoids in artichoke extracts by HPLC (350 nm). For peak assignment see Table 1.

HPLC separations highlighted the presence of 14 compounds (peaks in Figure 1) belonging to hydroxycinnamate and flavone groups (Table 1): the identification of some acid compounds (peaks 3–6 and 11, Figure 1) and flavonoids (peaks 8 and 12, Figure 1) was based on the comparison of their UV spectra and retention times with those of the corresponding standards.

Subsequent mass spectrometry analyses confirmed peak assignment and allowed further characterization of individual substances for all compounds. Mass spectrometric behavior (Table 1) was identified on the basis of pseudomolecular ion or fragmentation patterns in the MS and by comparing the elution order with that reported in the literature. In accordance with Schütz et al. [19], Romani et al. [9], Negro et al. [7], all the qualitative profiles were identified in each of the three artichoke head part analysed. Caffeic acids, named also hydroxycinnamic acids, are composed of a wide range of derivatives, of which chlorogenic and 1,5-O-dicaffeoylquinic acids are the most abundant components [4,19,20,21,22,23,24,25,26,27,28].

In a previous study, we demonstrated that nitrogen fertilization influenced the weight of the principal heads, dry matter, and SPAD index, moreover the application of biostimulant products influenced the characteristics of the principal and secondary heads [29]. In this study the analysis of variance of the globe artichoke polyphenolic content (Supplementary Table S1) showed that, on average, polyphenol content varied in relation to the year (Y) and head parts (HD), and in relation to the cultivar (C); little influence was observed for nitrogen (N) doses and biostimulants (B) foliar application.

### 2.1. Caffeoylquinic Acids Content

The identification of individual peaks of caffeoylquinic acids was based on a comparison of their UV spectra and retention times with those of standards utilized, whose sum constitutes the total.

As reported in Table 2, the contents of all the caffeoylquinic acids varied significantly between years (*p* ≤ 0.001) and head parts (*p* ≤ 0.001). The effect of the cultivar was less marked, showing a significant (*p* ≤ 0.05) change only for 3-caffeoylquinic acid, 5-caffeoylquinic acid and 1,5-di-caffeoylquinic acid. No significant variation was observed for nitrogen fertilization and biostimulating products.

The content of all caffeoylquinic acids was higher in 2017, with the exception of the 1,3-di-caffeoylquinic acid, possibly due to the different ages of the analyzed tissues and to different climatic conditions of the cultivation year. For the same reasons, the content of all caffeoylquinic acids was highly significant for all interactions (Table S1).

Total caffeoylquinic acids were more abundant in cv. Opal (25,597.90 mg kg^−1^) compared to cv. Madrigal (18,265.06 mg kg^−1^) (*p* = 0.0437). In addition, their content in different parts of the head increased from the outer bracts to the receptacle, and then from senile to juvenile tissues, with different behavior in cultivars, as confirmed by the significance of “CxHP” interaction (*p* = 0.0368) (Table S1), and as already observed for CGA in other globe artichoke varieties by Sonnante et al. [5].

As showed in Table 2, total content of caffeoylquinic acids detected in various head portions of cv. Opal varied in relation to the dose of N and increased by 30% in the fertilized theses treated with biostimulants. No increase was observed in the non-fertilized thesis but treated only with biostimulants, which produced even a decrease of the total caffeoylquinic acids content.

The higher content of total caffeoylquinic acids detected in the heads of the cultivar Madrigal was found at a dose of 100 kg ha^−1^, in the thesis not treated with biostimulating products.

In non-fertilized theses, a growing content in caffeoylquinic acids was observed passing from senescent to juvenile tissues; in the same theses, the addition of biostimulants produced an increase of the concentrations in all tissues except in the receptacle, whose quantity was reduced by about 30%.

The characterization of phenolic compounds between outer bracts and heads was compared by Romani [9] on two globe artichoke accessions. They reported 1,5-dicaffeoylquinic acid as the most abundant compound. Higher amounts of chlorogenic acid was also found by Fratianni et al. [8] and Sonnante et al. [5] in the inner bracts of three (Tondo di Paestum, Bianco di Pertosa and Violet de Provence) or two (Locale di Mola, S. Erasmo) different genotypes of globe artichoke, respectively. Analogous data have been reported by Pandino et al. [26] who found such compounds at higher amounts in the artichoke receptacles than in the outer bracts.

### 2.2. Flavonoids: Luteolin and Apigenin Content

The average quantities of each individual luteolin are shown in Table 3. Significant variations were observed between years, parts of heads, biostimulants, and cultivar.

The concentration of luteolin-rutinoside, luteolin-glucoside, and total luteolin increased when passing from external bracts to receptacle.

The effect of the cultivar was less marked for luteolin-glucoside, being significant only for luteolin-rutinoside (*p* ≤ 0.001), with cv. Opal containing 37% more than cv. Madrigal, and for luteolin (*p* ≤ 0.05) with cv. Madrigal containing 51% more than cv. Opal.

Nitrogen fertilization did not significantly influence any of the examined flavonoids while the effect of the biostimulants was significant for luteolin-rutinoside content: the most abundant of the luteolin family (approximately 70% of the total) increased by about 20% with the application of biostimulating products at the dose of 3 L ha^−1^. In the untreated control (without fertilization and application of biostimulating) the content of luteolin-rutinoside grow from senescent to younger tissues in both cultivars; a similar trend was found in the same thesis treated with biostimulants, except for the receptacle.

A higher content of total luteolin was observed in the year 2017 (39,178.40 mg kg^−1^), with the exception only of luteolin-glucuronide (8.50 mg kg^−1^) and luteolin (371.82 mg kg^−1^), for which the major quantity was found in 2016.

As well as for the caffeoylquinic acids, total luteolin content (*p* ≤ 0.001) increased when passing from outer bracts towards the receptacle for both cultivars: this was also confirmed by the significance of the “CxHP” interaction (*p* = 0.0165).

Total apigenin content varied significantly between years (*p* ≤ 0.001) and amongst the head parts (*p* ≤ 0.05), as showed in Table 4. Unlike other polyphenols, the content of all individual apigenins was greater in 2016. With regard to the various head parts, apigenin 7-O glucoside and apigenin 7-O glucuronide showed the highest concentrations in the external bracts, which strongly reduced in intermediate (13.19 and 18.84 mg kg^−1^), and subsequently increased in the internal bracts (16.15 and 23.07 mg kg^−1^) and receptacle (21.43 and 30.62 mg kg^−1^). The apigenin-rutinoside increased from outer bracts to the receptacle (from 7.26 mg kg^−1^ to 25.13 mg kg^−1^). The artichoke phenolic content is related to the plant age. In general, immature head tissues have higher phenol contents than mature heads, as total polyphenols, detected in different cultivars of *C. cardunculus* var. *scolymus*, increased from external to internal parts [26].

Interactions of the effects of nitrogen fertilization and biostimulating products on cultivars showed no significance influence on apigenine concentrations (Table S1).

A study by Schütz et al. [19] on globe artichoke heads identified apigenin-7-Oglucuronide, luteolin-7-O-rutinoside, and luteolin-7-O-glucoside as the main flavonoids. Pandino et al. [25] analyzed and compared the phenolic profile between capitula of wild cardoon, globe artichoke and cultivated cardoon. They reported apigenin and its 7-O-glucuronide as predominant in the cultivated plant, thus confirming their potential use as a source of such flavonoids.

Romani et al. [9] studied in detail all phenolic compounds present in Violetto di Toscana and Terom artichokes and demonstrated that flavonoids are present at higher amounts in leaves followed by heads, while stems are completely devoid of flavonoids. Finally, the capitula of artichoke is a natural source of apigenins, one of the most important flavonoids that have several biological and pharmacological activities.

As said above, the globe artichoke is rich in polyphenol compounds that play simultaneously the functions of nutrition and pharmaceuticals on the human body.

In agreement with D’Antuono et al. [30], in our study we found that the qualitative composition of artichoke polyphenols can be attributed to hydroxycinnamic acids and flavonoids: 5-O-caffeoylquinic acid and 1,5-O-dicaffeoylquinic; apigenin and luteolin (both present as glucosides and rutinosides), the most abundant components.

A significant influence of the genotype on both total polyphenol content and individual compounds has been demonstrated, as previously observed for other crops such as strawberry [31], fig [32], potato, and some stone fruits [33,34].

This study highlights that cv. Opal is richer in luteolin, chlorogenic acid and 4-O-caffeoylquinic acid than cv. Madrigal, probably due to higher isomerization reaction of cynarin (1,3-di caffeoylquinic acid) in the head tissues, in agreement with Schütz et al. and Lombardo et al. [19,21]. On the contrary, Madrigal is an interesting source of apigenin and its derivatives. As reported previously, these compounds are probably the major factors of the antispasmodic and anti-inflammatory activity attributed to polyphenols [1,35,36], due to their antioxidant activity [37,38] and their good absorption after ingestion [39,40]. Apigenin and other flavonoids are not very common in food plants [41] and therefore the artichoke could represent a significant source of these bioactive compounds.

According to our results, cv. Opal, which possesses the highest amount of polyphenols, could be suitable for fresh market or industrial extraction of natural antioxidants. Cv. Madrigal, characterized by a lower content of polyphenols in the different head parts, could be more appropriate for the canning industry, due to the more reduced susceptibility to the browning of tissue during handling and storage [1,42,43].

The level of phenolic compounds in different cultivars, although under genetic control, may be subject to significant fluctuations in relation to physiological changes during the plant growth, weather conditions and adopted agronomic practices.

Polyphenol content, in the head at commercial maturity, increases about 30% passing from the external bracts towards the internal ones and the receptacle, from senile to juvenile tissues in agreement with previous studies [7,8,44,45]. In particular, it is interesting to note that caffeoylquinic acids play an important role in tissues lignification [46] and their decrease in older bracts could be explained as they may be used as precursors for the biosynthesis of lignin. The outer bracts, in fact, are rich in lignin since they also provide physical resistance to the internal parts of the head, defending them from biotic and abiotic stress.

The results obtained for the receptacle show that it is the richest part in phenolic compounds and highlight the importance of this vegetable as a functional food, since the receptacle represents a great part of the edible portion of the artichoke head. Moreover, outer bracts, which are generally discarded, could become an unexpected source of nutraceutical compounds and a source of income for farmers and industry [21,47,48].

Fratianni et al., Lombardo et al., Pandino et al., Alamanni et al., Blanco et al. and Curadi et al. [8,20,21,25,49,50] showed that polyphenolic concentration, apart from genotypes, can be attributed to different agronomic management as well as experimental procedures or climatic conditions.

Our results showed that different agro-climatic conditions recorded during winter in the second year, which was characterized by lower temperatures and higher rainfalls than the previous one, were presumably more favorable for the biosynthesis of polyphenolic compounds. This is in accordance with [4] who found higher amounts of polyphenols in artichoke heads collected in October compared to those collected in September. Moreover, other studies demonstrated that environmental stresses can play considerable influence on the levels of secondary metabolites, such as polyphenols, in plants [51,52].

With regard to fertilization, apart from the cultivar analyzed, the highest content of caffeoylquinic acids and luteolin is obtained by using an ammonium nitrate dose of 100 kg ha^−1^. On the contrary, total apigenin seems to be more synthesized in the absence of fertilization even though among the fertilized thesis, the dose of 100 kg ha^−1^ increased the synthesis of these bioactive compounds. The application of biostimulants has also encouraged the accumulation of polyphenols in artichoke heads.

A different response to treatments was observed for both the cultivars analyzed. Our results on nitrogen effects are generally in agreement with [50] who argued that increases in nitrogen fertilization influence in reverse the contents of bioactive substances in artichoke, concluding that doses between 80 and 120 kg ha^−1^ of nitrogen fertilizer represent a point of balance between the quantity and the quality of artichoke in open field cultivation. Higher yields and improvement of the quality of products have often been attributed to the appropriate contribution of nutriments, and mineral nutrient application becomes essential to satisfy nutrient uptake.

Finally, the results are consistent with what is reported in the literature for artichoke and other plant species: the quantities of polyphenols in artichoke heads vary depending on the cultivar, environmental conditions, and phenological phase [1,4,7,49,53,54,55,56].

## 3. Materials and Methods

Two-year field-experiments were carried out in the same field trial during the growing seasons 2015–2016 and 2016–2017.

The field experiments on Opal and Madrigal artichoke cultivars (Nunhems Brand, BASF Seeds) were conducted at the Experimental Station of CNR-Institute of Biosciences and Bioresources, in Metapontino Plain, (40°10′ N–16°39′ E), Policoro, Italy. The trial field is located around 15 m above the sea level and is characterized by a sub-humid climate according to the De Martonne classification [24].

We compared four increasing doses of nitrogen (0, 50, 100 and 150 kg ha^−1^) supplied as ammonium nitrate (NH_4_NO_3_) 34% N and two doses of biostimulants (0 and 3 L ha^−1^), which were applied in combination by foliar spraying, based on seaweed cream and microelements. Nitrogen fertilizations and foliar spraying of biostimulants were applied seven times during all the crop production cycle at the programmed doses: every twenty days starting from fifty days after transplantation or regrowth. The biostimulants used were commercially identified as “BM 86” and “GA EU”, seaweed cream (made of Ascophyllum nodosum and oligosaccharides) and trace elements, produced and sold by Goëmar (Goëmar Saint-Malo, France), as plant physiological activator; the stimulants are suggested by the company to activate nutrition, promote flowering, secure/optimize fruits setting and give a better contribution to the “fruits” of vegetables, leading to a faster (and more homogenous) development. The stimulants suggest the activation of several physiological functions of the plant such as nutritional pathways and biosynthesis of flowering promotion.

In all experiments, foliar nutrition was applied in the same combination of types and concentrations of fertilizers and on the same dates.

### 3.1. Analysis of Polyphenols

Analysis of polyphenol content in artichoke heads was carried out on fresh plant material. In agreement with the method of [7], heads were harvested at the commercial maturity: external (ExB, 9–10 bracts), intermediate (ImB, 9–10 bracts) and internal (InB, remaining bracts) bracts as well as receptacles (Rec) were manually separated and immediately frozen at −20 °C. Tissues were collected from three samples of each experimental plot considered in this study for each of the two field trial years (2015–2016 and 2016–2017).

#### 3.1.1. Reagents and Solvents

Solvents were purchased from J.T. Baker (Deventer, Holland) and were of analytical or High-Performance Liquid Chromatography (HPLC) grade. Deionized water was used for all the analyses. 5-O-caffeoylquinic acid (CGA), caffeic acid, apigenin 7-O-glucoside and luteolin 7-O-glucoside (cynaroside) were obtained from Extrasynthèse (Lyon, France); 1,3-di-O-caffeoylquinic acid (cynarin Echinacea) and 1,5-di-O-caffeoylquinic acid (cynarin Artichoke) were from ChromaDex Inc. (Santa Ana, CA, USA). Formic acid and HPLC grade water were purchased from J.T. Baker (Milan, Italy). LC-MS grade solvent acetonitrile was purchased from Sigma-Aldrich (Milan, Italy).

#### 3.1.2. Extraction Procedure

Polyphenol extraction was performed according to Negro et al. methodology [7] from the above mentioned tissues and repeated in triplicate for each of the two growing years. Briefly, 0.5 g of each sample were incubated for 1 h at 4 °C in 5 volumes of acetone:ethanol:methanol (70:15:15). The supernatant was collected and the residue was resuspended in 5 volumes of ethyl acetate and incubated at 4 °C for 1 h. The 2 supernatants were separately concentrated to dryness in a Rotavapor 144 R (Büchi, Switzerland) and re-suspended in 1 mL methyl alcohol before being pooled together and subjected to polyphenol characterization.

#### 3.1.3. HPLC-DAD Analysis

HPLC analyses were carried out by using Beckman-Coulter (Fullerton, CA, USA) HPLC System Gold chromatograph equipped along with a DAD programmable detector (System Gold, series 166) operated by a 32 Karat software package (Beckman-Coulter). A reversed phase column Luna C18 (250 × 4.6 mm i.d., particle size 5 μm; Phenomenex, Torrance, CA, USA) was used.

The following gradient system was used with water (containing 0.01% trifluoroacetic acid; solvent A) and 95% acetonitrile (containing 0.01% trifluoroacetic acid; solvent B): 0 min, 90% A-10% B; 30 min, 50% A-50% B; 35 min, 0% A-100% B. The flow was maintained at 1 mL/min and the injection volume was 10 μL. Diode array detection was between 200 and 600 nm, and absorbance was recorded at 310 and 350 nm.

#### 3.1.4. Qualitative and Quantitative Analysis

Compound identification was achieved by combining different information: positions of absorption maxima (λ max), retention times (min), mass spectra and the corresponding daughter MS-MS fragments were compared with those from pure standards and/or interpreted with the help of structural models already hypothesized in the literature [19,25,26].

Quantification was made by using six points (10–1000 ppm) calibration curves with the standards of 5-O-caffeoylquinic acid (chlorogenic acid, CGA), 1,3-di-O-caffeoylquinic acid, 1,5-di-O-caffeoylquinic acid, caffeic acid, apigenin 7-O-glucoside, luteolin 7-O-glucoside, with R2 values ranging between 0.9997 and 0.9999. Monocaffeoylquinic acids were calculated as CGA. Apigenin and luteolin derivatives were calculated as apigenin 7-O-glucoside and luteolin 7-O-glucoside, respectively [19].

### 3.2. HPLC-DAD-ESI-MS/MS Analysis

The HPLC-DAD-MS/MS system consisted of a capillary HPLC 1290 Infinity (Agilent Technologies, Palo Alto, CA, USA) equipped with a binary pump solvent delivery, thermostatic column compartment, diode array detector and a QQQ 6410 mass detector (Agilent Technologies) coupled with a pneumatic nebulizer-assisted electrospray LC-MS interface. A Poroshell column 120 EC-C18 (150 × 2.1 mm i.d., particle size 2.7 μm, Agilent Technologies) was used, with a pre-column Gemini C18 (4 × 2 mm i.d., particle size 5 μm; Phenomenex). The following gradient system was used with acetonitrile (solvent A) and water/formic acid (99:1, *v*/*v*) (solvent B): 0 min, 5% A-95% B; 10 min, 13% A-87% B; 20 min, 15% A-85% B; 30 min, 22% A-78% B; 50 min 22% A-78% B; 55 min 5% A-95% B; stop time to 70 min. The flow was maintained at 0.2 mL/min; sample injection was 3 μL. Diode array detection was between 200 and 600 nm, and absorbance was simultaneously recorded at 350 nm (luteolin derivatives), 330 nm (apigenin derivatives), 320 nm (hydroxycinnamic acid).

Negative electrospray mode was used for ionization of molecules with capillary voltage at 4000 V. The fragmentor voltage was 140 V, and the collision voltage was 15 V. Nitrogen was used both as drying gas at a flow rate of 9 L min^−1^ and as nebulizing gas at a pressure of 40 psi. Temperature of drying gas was 350 °C. In the full scan (MS) and product ion (MS/MS) modes, the monitored mass range was from *m/z* 100 to 1200. Typically, two runs were performed during the HPLC-ESI-MS analysis of each sample. First, an MS full-scan acquisition was performed to obtain preliminary information on the predominant *m/z* ratios observed during the elution. An MS/MS full-scan acquisition was then performed: Quadrupole 1 filtered the calculated *m/z* of each compound of interest, whilst Quadrupole 3 scanned for ions produced by nitrogen collision of these ionized compounds in the chosen range at a scan time of 500 ms/cycle. All data were acquired and processed using Mass Hunter software (version B.01.04; Agilent Technologies).

### 3.3. Statistical Analyses

The adopted experimental scheme was a split-plot, in which cultivars and nitrogen, in factorial combination, were the factors in the main plot, while the sub-plot factors were the two levels of biostimulants. Head parts (ExB, ImB, InB, and Rec) were considered as sub-sub-plot factor.

All collected data were subjected to statistical analysis of variance (Proc GLM) using adequate SAS options (Statistical Analysis System—SAS Institute, Cary, NC, USA, ver. 9.1.3, service pack 4). Performing factorial analysis, cultivar and nitrogen were considered at the same hierarchical level, then the influence of biostimulant as sub-plot factor was tested.

In order to assess the repeatability of the observed behavior over the years, the analysis was performed considering the year as a random variable and testing the significance of the main factors on the interaction “year × treatment” [27].

Differences between the means were compared using the SNK (Student-Newman-Keuls) test at a level of probability of 0.05.

## 4. Conclusions

A two-year investigation aimed at assessing the effect of different doses of nitrogen fertilization and application of biostimulants on the quality of two artichoke cultivars (Opal and Madrigal), showed that the synergic action of these compounds is effective in improving some aspects of head quality.

In cv. Opal, caffeoylquinic acids and luteolins were more abundant in correspondence with the dose of fertilization of 100 kg ha^−1^ of ammonium nitrate, while apigenins were not affected by nitrogen fertilization; in cv. Madrigal, caffeoylquinic acids were synthesized mainly at the dose of nitrogen fertilization of 100 kg ha^−1^, instead luteolin and apigenin were not affected by nitrogen fertilization. In any case, the application of biostimulants promoted the accumulation of polyphenols in artichoke heads.

Although the level of phenolic compounds in the heads is genetically controlled and is related to head parts, it may be subject to significant variations in relation to the weather conditions and the adopted agronomic practices, such as nutrition, irrigation and pest control.

The results here obtained confirmed those previously reported in the literature, and highlighted the positive aspects of the synergistic effect of nitrogen fertilization in addition of biostimulants foliar application.

Further studies are needed to better understand interactions exerted by agronomic and climatic factors on the accumulation of nutraceutical compounds in the artichoke head.

## Figures and Tables

**Table 1:** Caffeoylquinic acids and flavones in artichoke: their UV Spectra and characteristic ions as determined by HPLC-DAD-MS/MS analysis.

No.	Compound	Retention Time (min)	HPLC-DAD λ_max_ (nm)	[M-H]^− *a*^ *m*/*z*	MS/MS *^b^* *m*/*z*	Comparison with Standard
1	1-O-Caffeoylquinic acid	6.21	233, 305sh, 328	353	191	no
2	3-O-Caffeoylquinic acid	8.65	241, 303sh, 325	353	191	no
3	5-O-Caffeoylquinic acid	10.48	241, 305sh, 326	353	191	yes
4	4-O-Caffeoylquinic acid	11.51	236, 303sh, 326	353	191	no
5	Caffeic acid	12.30	237, 302sh, 323	179	135	yes
6	1,3-di-O-Caffeoylquinic acid	13.59	242, 307sh, 322	515	353	yes
7	Luteolin 7-O-rutinoside	25.01	256, 266sh, 350	593	285	no
8	Luteolin 7-O-glucoside	26.13	253, 266sh, 347	447	285	yes
9	Luteolin 7-O-glucuronide	26.88	254, 268sh, 343	461	285	no
10	Apigenin 7-O-rutinoside	29.32	249, 304sh, 328	577	269	no
11	1,5-di-O-Caffeoylquinic acid	29.97	243, 303sh, 329	515	353	yes
12	Apigenin 7-O-glucoside	30.74	229, 266, 339	431	269	yes
13	Apigenin 7-O-glucuronide	31.76	267, 335	445	269	no
14	Luteolin	43.74	254, 266sh, 347	285		no

*^a^*(M-H)^−^, deprotonated molecular ion; *^b^* production.

**Table 2:** Effects of treatments (year, cultivar, rate of N and biostimulants, and head part) on caffeoylquinic acids content (mg kg^−1^ FM) of two globe artichoke cultivars.

	1-Caffeoylquinic Acid	3-Caffeoylquinic Acid	4-Caffeoylquinic Acid	5-Caffeoylquinic Acid	Caffeic Acid	1,3-Dicaffeoylquinic Acid	1,5-Dicaffeoylquinic Acid	Total
Year	**	**	**	**	**	**	**	**
2016	11.99 ^b^	7.69 ^b^	16.91 ^b^	255.09 ^b^	43.94 ^b^	13.01 ^a^	16,781.58 ^b^	17,130.22 ^b^
2017	18.71 ^a^	9.16 ^a^	35.54 ^a^	531.52 ^a^	80.51 ^a^	5.62 ^b^	26,051.68 ^a^	26,732.74 ^a^
Cultivar		*		*			*	*
Opal	19.05	9.26 ^a^	32.08	497.25 ^a^	60.92	9.00	24,970.34 ^a^	25,597.90 ^a^
Madrigal	11.65	7.58 ^b^	20.38	289.36 ^b^	63.54	9.64	17,862.91 ^b^	18,265.06 ^b^
Nitrogen Rate (kg/ha)								
0	14.44	8.39	31.05	418.12	66.72	9.62	24,528.32	25,076.66
50	13.02	8.29	22.109	302.93	56.44	9.06	17,974.98	18,386.80
100	16.81	8.94	29.27	503.20	65.44	10.09	24,573.34	25,207.09
150	17.14	8.09	22.49	348.97	60.31	8.51	18,589.87	19,055.38
Biostimulating Rate (L/ha)								
0	15.98 ^a^	8.02 ^b^	24.68 ^b^	356.27 ^b^	56.79 ^b^	10.36 ^a^	18,720.40 ^b^	19,192.51 ^b^
3	14.72 ^b^	8.82 ^a^	27.78 ^a^	430.34 ^a^	67.66 ^a^	8.28 ^b^	24,112.85 ^a^	24,670.46 ^a^
Head Parts	**	**	**	**	**	**	**	**
External Bracts	11.08 ^d^	7.70 ^c^	13.71 ^d^	174.93 ^d^	33.30 ^d^	1.07 ^d^	8504.33 ^d^	8746.12 ^b^
Intermediate Bracts	17.76 ^b^	8.95 ^b^	31.30 ^b^	437.54 ^b^	78.79 ^b^	7.77 ^c^	21,017.42 ^c^	21,581.54 ^c^
Inner Bracts	14.04 ^c^	7.70 ^c^	32.90 ^a^	533.54 ^a^	56.21 ^c^	15.03 ^a^	27,092.25 ^b^	27,753.79 ^b^
Receptacle	18.52 ^a^	7.22 ^d^	27.00 ^c^	427.22 ^c^	98.61 ^a^	13.40 ^b^	29,052.51 ^a^	29,644.49 ^a^

Within each column in year, cultivar, nitrogen and biostimulants rates, and head parts, followed by a different letter, values are significantly different according to SNK test (when two comparisons are shown).*, ** = Significant at the *p* < 0.05, 0.01 probability levels, respectively. Empty cell = not significant.

**Table 3:** Effects of treatments (year, cultivar, rate of N and biostimulants, and head part) on luteolines (mg kg^−1^ FM) in two globe artichoke cultivars.

	Luteolin Rutinoside	Luteolin Glucoside	Luteolin Glucuron	Luteolin	Total
Year	**	**	**	**	**
2016	22,009.10 ^b^	7761.27 ^b^	8.94 ^a^	371.82 ^a^	30,151.10 ^b^
2017	24,667.50 ^a^	14,279.03 ^a^	8.50 ^b^	223.30 ^b^	39,178.40 ^a^
Cultivar	**			*	
Opal	28,728.70 ^a^	9139.41	10.17	195.18 ^b^	38,073.40
Madrigal	17,947.90 ^b^	12,900.88	7.28	399.96 ^a^	31,256.00
Nitrogen Rate (kg/ha)					
0	27,686.20	13,480.50	12.66	384.49	41,563.90
50	21,349.30	6383.00	6.70	327.38	28,066.30
100	27,377.80	18,994.10	8.70	196.08	46,576.70
150	16,940.00	5222.90	6.84	282.32	22,452.10
Biostimulating Rate (L/ha)	*				
0	21,001.90 ^b^	13,786.66 ^a^	8.16 ^b^	289.21 ^b^	35,085.90
3	25,674.70 ^a^	8253.64 ^b^	9.29 ^a^	305.92 ^a^	34,243.60
Head Parts	**	**			**
External Bracts	5529.50 ^d^	2514.60 ^c^	6.12	215.37	8265.60 ^d^
Intermediate Bracts	22,932.80 ^c^	2200.90 ^d^	7.52	367.53	25,508.80 ^c^
Inner Bracts	34,658.60 ^a^	9968.40 ^b^	8.07	348.33	44,983.40 ^b^
Receptacle	30,232.30 ^b^	29,396.70 ^a^	13.18	259.03	59,901.20 ^a^

Within each column, letters following values refer to significant differences according to SNK test (when two comparisons are shown). *, ** = Significant at the *p* < 0.05, 0.01 probability levels, respectively. Empty cell = not significant.

**Table 4:** Effects of year, cultivar, rate of N and biostimulants, and head part on apigenin (mg kg^−1^ FM) in two globe artichoke cultivars.

	Apigenin Rutinoside	Apigenin 7-O Glucos	Apigenin 7-O Glucur	Total
Year	**	**	**	**
2016	17.11 ^a^	29.92 ^a^	42.74 ^a^	89.78 ^a^
2017	11.53 ^b^	24.58 ^b^	35.12 ^b^	71.23 ^b^
Cultivar				
Opal	17.72	22.69	32.41	72.82
Madrigal	10.92	31.82	45.45	88.19
Nitrogen Rate (kg/ha)				
0	24.01	41.55	59.36	124.91
50	10.98	20.88	29.83	61.69
100	13.99	22.39	31.98	68.36
150	8.29	24.20	34.57	67.07
Biostimulating Rate (L/ha)				
0	12.65	24.83	35.47	72.96
3	15.99	29.67	42.39	88.06
Head Parts	**	*	*	*
External Bracts	7.26 ^d^	58.25 ^a^	83.21 ^a^	148.72 ^a^
Intermediate Bracts	9.05 ^c^	13.19 ^d^	18.84 ^d^	41.07 ^d^
Inner Bracts	15.84 ^b^	16.15 ^c^	23.07 ^c^	55.06 ^c^
Receptacle	25.13 ^a^	21.43 ^b^	30.62 ^b^	77.18 ^b^

Within each column, letters following values refer to significant differences according to SNK test (when two comparisons are shown). *, ** = significant at the *p* < 0.05, 0.01 probability levels, respectively. Empty cell = not significant.

## Data Availability

The data presented in this study are available on request from the corresponding author.

## Supplementary Materials

The following are available online at https://www.mdpi.com/article/10.3390/plants11152067/s1, Table S1: Complete scheme of analysis of variance and of the interaction between all the main factors on the polyphenols content in artichoke heads.
