# Supplementary material for: Polyphenolic Compound Variation in Globe Artichoke Cultivars as Affected by Fertilization and Biostimulants Application
Source: Plants (Basel). 2022 Aug 8;11(15):2067. doi: 10.3390/plants11152067 (PMC9370648; doi:10.3390/plants11152067)
Supplement: Supplementary file 1 [file plants-11-02067-s001.zip › plants-1816796-supplementary.pdf]

Supplementary Table S1. complete scheme of analysis of variance and of the interaction between all the main factors on the polyphenols content in artichoke heads.

	**1-Caffeoyl quinic acid**	**3-Caffeoyl quinic acid**	**4-Caffeoyl quinic acid**	**5-Caffeoyl quinic acid**	**Caffeic Acid**	**1,3-Dicaffeo ylquinic acid**	**1,5-Dicaffeo ylquinic acid**	**TOTAL Caffeoyl quinic acid**	**Luteolin rutinoside**	**Luteolin glucoside**	**Luteolin glucuron**	**Luteolin**	**TOTAL Luteolin**	**Apigenin rutinoside**	**Apigenin 7-O glucos**	**Apigenin 7-O glucur**	**TOTAL Apigenin**
Year (Y)	**	**	**	**	**	**	**	**	**	**	**	**	**	**	**	**	**
Cultivar (C)		*		*			*	*	**			*					
Nitrogen (N)																	
Biostimul. (B)									*								
Head Parts (HP)	**	**	**	**	**	**	**	**	**	**			**	**	*	*	*
Y*C	**	**	**	**	**	**	**	**		**	**	**		**	**	**	**
Y*N	**	**	**	**	**	**	**	**	**	**	**	**	**	**	**	**	**
Y*HP	**	**	**	**	**	**	**	**	**	**	**	**	**	**	**	**	**
Y*B	**	**	**	**	**	**	**	**		**	**	**	**	**	**	**	**
C*HP	**	*		**			*	*	**	**		*	*	*			
C*B						*					*						
N*HP				**		*	*	*	**					**			
N*B									**			*			**	**	*
B*HP		*		*			**	**	**								
C*N*Y	**	**	**	**	**	**	**	**	**	**	**	**	**	**	**	**	**
C*B*Y	**	**	**	**	**	**	**	**		**	**	**	**	**	*	*	**
C*HP*Y	**	**	**	**	**	**	**	**	**	**	**	**	**	**	**	**	**
N*B*Y	**	**	**	**	**	**	**	**	**	**	**	**	**	**	**	**	**
N*HP*Y	**	**	**	**	**	**	**	**	**	**	**	**	**	**	**	**	**
B*HP*Y	**	**	**	**	**	**	**	**	**	**	**	**	*	**	**	**	**
C*N*B				*								*					
C*N*HP		*					**	**	**					**			
C*B*HP	*	*		**		*				*			*	*	*	*	*
N*B*HP		*		*			**	**	**								
C*N*B*Y	**	**	**	**	**	**	**	**	**	**	**	**	**	**	**	**	**
C*N*HP*Y	**	**	**	**	**	**	**	**	**	**	**	**	**	**	**	**	**
C*HP*B*Y	**	**	**	**	**	**	**	**	*	**	**	**	**	**	**	**	**
HP*N*B*Y	**	**	**	**	**	**	**	**	**	**	**	**	**	**	**	**	**
C*N*B*HP				**	*		**	**	**								

*, ** = Significant at $P < 0.05$, $P < 0.01$ probability levels, respectively. Empty cell=not significant
